# Spontaneous mediastinal haemorrhage linked with thymic carcinoma and myelodysplasia: a case report

**DOI:** 10.4076/1757-1626-2-7821

**Published:** 2009-06-09

**Authors:** Suresh R Fernando, Filip Van Tornout, Richard Y Ball, Jennie Z Wimperis

**Affiliations:** 1 Departments of Thoracic Surgery, Norfolk and Norwich University Hospitals NHS Foundation TrustNorwichUK; 2 Histopathology/Cytopathology, Norfolk and Norwich University Hospitals NHS Foundation TrustNorwichUK; 3 Haematology, Norfolk and Norwich University Hospitals NHS Foundation TrustNorwichUK

## Abstract

We report an unusual sequence of clinico-pathological manifestations of myelodysplastic syndrome and thymic squamous cell carcinoma. A 77-year-old man with a two-month history of myelodysplastic syndrome was admitted with acute chest pain and shortness of breath. Radiological investigations revealed an anterior mediastinal mass, associated with mediastinal haemorrhage. The mass was excised via a standard median sternotomy and was found to be an infiltrating squamous cell carcinoma, which arose from a multilocular thymic cyst.

## Introduction

Spontaneous mediastinal haemorrhage is rare, usually presenting with chest pain and dyspnoea. In this paper, we describe a patient who developed haemorrhage into a multilocular thymic cyst, which showed focal development of squamous cell carcinoma. This thymic lesion was associated with a form of myelodysplasia (Refractory anaemia with excess blasts-1: RAEB-1), progressing to myeloid sarcoma. It ultimately proved fatal.

Myelodysplastic syndrome (MDS) is a group of clonal haematopoietic stem cell disorders characterised by marrow dysplasia and ineffective haematopoiesis in one or more of the myeloid lines, resulting in low blood cell counts [1]. The median age at diagnosis of MDS is 70 years, and the condition is more prevalent in males. RAEB-1 is a form of MDS in which there are 5-9% myeloblasts in the bone marrow or 2-4% blasts in the peripheral blood. It is characterised by progressive anaemia and a tendency to develop acute myeloid leukaemia in 25-33% of cases; the rest of the patients die of the consequences of marrow failure [1]. Overall survival is about 9-16 months. Although most patients with MDS have no obvious cause for their disease, there are well-established risk factors, including: smoking; ionising radiation; and chemotherapy for other malignancies.

Myeloid sarcoma is a tumour mass of myeloblasts or immature myeloid cells occurring in an extramedullary site or in bone [1]. It is associated with acute myeloid leukaemia or, in the case of MDS, it may be the first manifestation of blastic transformation.

## Case presentation

A 77-year-old white Caucasian man was admitted with a three-day history of chest pain, dyspnoea, sweating and haemoptysis. He was already under haematological review, having been diagnosed with MDS (RAEB-1). A bone marrow aspirate was hypercellular. It showed erythroid hyperplasia with prominent non-megaloblastic dyserythropoiesis, active left-shifted granulopoiesis with marked dysgranulopoiesis, and some diminutive megakaryocytes. No ring sideroblasts were seen. Myeloid blast cells were 9%, and no Auer rods were seen. The myeloid:erythroid cell ratio was 0.8:1. Lymphocytes were reduced in number, and there were fewer than 10% plasma cells. Cytogenetics studies showed trisomy 8, in keeping with MDS. No other clonal chromosome abnormalities were detected. He had started blood and platelet transfusions 6 weeks prior to this acute admission.

Examination on admission showed mild sinus tachycardia (pulse rate = 91/min) and hypotension (100/70 mmHg), the jugular venous pressure was not raised and auscultation of the heart was normal. The trachea was in the midline and the respiratory rate was 22/min. Auscultation of the lungs revealed basal crepitations and reduced air entry at the right base.

Haematological investigations showed a haemoglobin of 9.1 g/dL and thrombocytopenia (22 × 10^9^/L; N = 150-400 × 10^9^/L), with a normal leukocyte count (5.3 × 10^9^/L; N = 4.0-11.0 × 10^9^/L*)*. The prothrombin and activated partial thromboplastin times were within normal limits. Basic biochemical tests, including urea and electrolytes, liver function tests and cardiac enzymes, showed no significant abnormality.

Chest X-ray showed a widened mediastinum. Computed tomographic pulmonary angiography (CTPA) revealed a mass of soft tissue density in the anterior mediastinum, associated with mediastinal haemorrhage and bilateral small pleural effusions. The CTPA also confirmed a previously noted infra-renal aortic aneurysm. A magnetic resonance imaging (MRI) scan of the chest located the mediastinal mass in relation to, but outside, the pericardium (Figure). There were foci of high signal suggestive of haemorrhage but also some low signal components on T1 and T2, suggesting the possibility of fibrous tissue.

**Figure 1.**
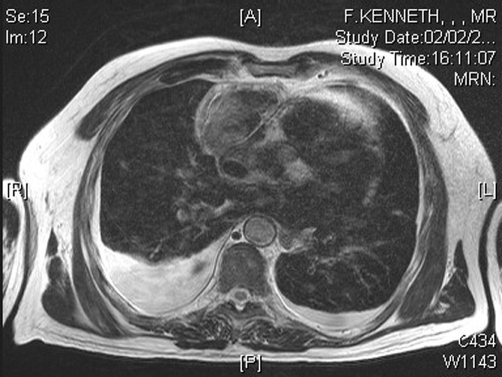
MRI scan showing the anterior mediastinal mass with associated haemorrhage and bilateral pleural effusions.

The patient underwent total thymectomy via standard median sternotomy, once he was haemodynamically stable following platelet transfusion. At operation, the anterior mediastinal mass (measuring 10 × 7 × 3 cm) was stuck to pericardium anterolaterally on right and to the upper and middle lobes of the right lung. There were 700 ml of haemorrhagic fluid in the right pleural cavity. The thymus was resected with the anterior part of pericardium, wedge excisions of right upper and middle lobes and excision of right phrenic nerve and lymph nodes. His post-operative course was uneventful apart from developing a superficial sternal wound infection, which was treated with antibiotics.

Histopathological examination of the mediastinal mass revealed an infiltrating squamous carcinoma, which appeared to have arisen from a multilocular thymic cyst. The cysts, which were generally lined by an attenuated epithelium, but focally by well-differentiated squamous epithelium, were filled with blood clot showing focal organisation at the periphery. Adjacent thymic tissue showed haemorrhagic infarction, which appeared to be secondary to thrombosis of small vessels, at least one of which was an artery with a necrotic wall surrounded by a rim of squamous cell carcinoma. In the background was widespread focal proliferation of immature haematopoietic cells, predominantly myeloblasts and myelocytes. Three small lymph nodes were found within the tissue. All of them showed permeation of sinusoids by large malignant cells in keeping with metastatic squamous cell carcinoma and extramedullary haematopoietic activity similar to that seen in the thymic tissue. Cytological examination of the pleural fluid was negative for neoplasia.

He was followed up in thoracic surgical, haematological and oncological outpatient clinics, receiving supportive blood and platelet transfusion at regular intervals. Adjuvant radiotherapy was not offered in view of his MDS and other co-morbidities, including the large infra-renal aortic aneurysm. He was also seen by a vascular surgeon during this period, but the risks of aneurysmal repair were considered to outweigh the benefits.

Fourteen months after the initial surgery, he presented with a 12 × 7 × 3 mm dark red/brown soft tumour at the site of the sternal wound. It was excised under general anaesthesia and the histology was reported as myeloid sarcoma, which, given the preponderance of blasts, was regarded as a blastic variant of granulocytic sarcoma. Single fraction radiotherapy was given to the tumour site 4 weeks following excision.

Two months later, his general physical condition deteriorated and he was admitted for terminal palliative care, dying one day later.

## Discussion

Various paraneoplastic syndromes, myasthenia gravis being the commonest, have been described in association with thymic tumours [2]. There are a variety of other associations including: haematological abnormalities (e.g. pure red cell aplasia), malignancy (e.g. acute leukaemia) and other non-malignant manifestations, such as hypogammaglobulinaemia (Good's syndrome) [3]. According to Montresor and colleagues, 21% of patients that presented with thymoma were discovered to have other tumours [4].

Our case is unusual in two respects. One is its presentation with acute mediastinal and pleural haemorrhage. Presentation of thymic masses by mediastinal haemorrhage is most unusual, with few reports in the literature [5-7]. The bleeding into the tumour may have been triggered by arterial wall necrosis and thrombosis, compounded by the underlying haematological abnormality. The other, we believe, unique, aspect of our case is the association of thymic squamous cell carcinoma (arising from a multilocular thymic cyst) and MDS, which later progressed to myeloid sarcoma. Although thymic carcinoma and MDS are different clinical entities, it is legitimate to consider whether MDS might represent another form of thymic tumour-associated paraneoplastic state. Given the list of haematological associations noted above, it is a possibility, admittedly rare. If so, the underlying mechanisms remain to be elucidated.

## List of abbreviations

CTPA: Computed tomographic pulmonary angiography
MDS: Myelodysplastic syndrome
MRI: Magnetic Resonance Imaging
RAEB-1: Refractory anaemia with excess blasts-1
